# Arzneimittelinteraktionen bei kritisch Kranken

**DOI:** 10.1007/s00063-024-01214-z

**Published:** 2024-11-28

**Authors:** Romuald Bellmann, Stefan Weiler

**Affiliations:** 1 Arbeitsgruppe Klinische Pharmakokinetik, Labor für Inflammationsforschung, Gemeinsame Einrichtung Internistische Notfall- und Intensivmedizin, Universitätsklinik für Innere Medizin I, Medizinische Universität Innsbruck, Anichstraße 35, 6020 Innsbruck, Österreich; 2 Pharmakoepidemiologie, Institut für Pharmazeutische Wissenschaften, Eidgenössische Technische Hochschule (ETH) Zürich, Zürich, Schweiz

**Keywords:** Arzneimittelwechselwirkungen, Pharmakokinetik, Pharmakodynamik, Arzneimittelnebenwirkungen und unerwünschte Wirkungen, Metabolismus, Drug interactions, Pharmacokinetics, Pharmacodynamics, Drug-related side effects and adverse reactions, Metabolism

## Abstract

Bei kritisch Kranken besteht ein hohes Risko für unerwünschte Arzneimittelinteraktionen. Pharmakodynamische Interaktionen können Organtoxizität verstärken. Pharmakokinetische Interaktionen gründen meist auf einer Hemmung oder Induktion von Enzymen des Arzneimittelmetabolismus wie Cytochrom-P-450-Isoenzymen und Transporterproteinen wie P‑Glykoprotein. Inhibitoren dieser Moleküle können so toxische Wirkspiegel der entsprechenden Substrate herbeiführen, Induktoren hingegen subtherapeutische Konzentrationen. Amiodaron, Makrolide, Azol-Antimykotika, direkt wirksame Antikoagulanzien, Vitamin-K-Antagonisten, Immunsuppressiva, Rifampicin und einige ZNS-wirksame Substanzen sind besonders häufig an Interaktionen beteiligt. Eine Überprüfung der Medikation unter strenger Risiko-Nutzen-Abwägung, therapeutisches Drugmonitoring, Verwendung elektronischer Alert-Systeme und Datenbanken zusammen mit klinischer Bewertung können zur Vermeidung unerwünschter Arzneimittelinteraktionen beitragen.

## Abstract (en)

Critically ill patients are at high risk of adverse drug–drug interactions. Pharmacodynamic drug–drug interaction may cause organ damage. Pharmacokinetic interactions are usually caused by inhibition or induction of enzymes of drug metabolism such as cytochrome P-450 isoenzymes or transporter proteins such as P‑glycoprotein. Inhibitors of such molecules can cause toxic levels of the corresponding substrates, while inducers might produce subtherapeutic concentrations. Amiodarone, macrolides, antifungal azoles, direct-acting anticoagulants, vitamin K antagonists, immunosuppressants, rifampicin, and some central nervous system (CNS)-active substances are frequently involved in drug–drug interactions. Sound risk and benefit assessment of the applied medication, therapeutic drug monitoring, the use of electronic alert systems and databases along with clinical evaluation will contribute to avoiding adverse drug–drug interactions.

## Hintergrund

Bei kritisch Kranken umfasst die medikamentöse Therapie meist zahlreiche Wirkstoffgruppen und birgt daher ein hohes Risiko potenzieller Interaktionen [2]. Nicht jede mögliche Wechselwirkung führt tatsächlich zu einer unerwünschten Wirkung [23]. Besonders relevant sind Interaktionen, die die Gerinnung, die Nierenfunktion und die QTc-Zeit betreffen [23]. Der Schweregrad der resultierenden Komplikationen hängt von der zugrunde liegenden Interaktion und von zahlreichen individuellen Faktoren ab [23].

### Besonderheiten bei kritisch Kranken

Kritisch Kranke sind oft pathophysiologischen Veränderungen ausgesetzt, die die Pharmakokinetik und die Pharmakodynamik intensivmedizinisch relevanter Medikamente beeinflussen. So können in der Frühphase einer schweren Sepsis eine erhöhte Kapillarpermeabilität und die daraus resultierende Verschiebung intravasaler Flüssigkeit in den extravasalen Raum das Verteilungsvolumen wasserlöslicher Medikamente vergrößern. Eine in dieser Phase oft beobachtete hyperdyname Herzaktion mit erhöhtem Herzzeitvolumen und verstärkter renaler Perfusion kann eine glomeruläre Hyperfiltration bewirken. Beide Effekte können zu subtherapeutischen Konzentrationen hydrophiler Pharmaka führen. Kommt es in der Folge zu einer Verminderung des Herzzeitvolumens und einer Einschränkung der Nieren- und der Leberfunktion, resultieren daraus variable und eventuell toxische Wirkstoffspiegel. Bei Medikamenten, deren Dosis nicht nach der Wirkung titriert werden kann, wie Antibiotika, werden eine unzureichende Exposition oder eine Überdosierung erst mit Verzögerung sichtbar [4–6]. Therapeutisches Drugmonitoring hat bei diesen Wirkstoffen in der Intensivmedizin daher einen besonders hohen Stellenwert.

Therapeutisches Drugmonitoring hat in der Intensivmedizin einen hohen Stellenwert

Neben Medikamenteninteraktionen sind bei kritisch Kranken häufig Veränderungen der Pharmakokinetik durch extrakorporale Therapieverfahren, wie einer kontinuierlichen Nierenersatztherapie oder einer extrakorporalen Membranoxygenierung (ECMO), zu berücksichtigen, die – abhängig von dem Verfahren und von der Substanz – zu erheblichen Wirkstoffverlusten führen können. [4, 12, 16, 18, 24, 28].

## Klinische Relevanz

### Erhöhtes Risiko für unerwünschte Arzneimittelwirkungen

Für potenzielle Arzneimittelinteraktionen im Allgemeinen ergaben Studien eine Anzahl von 11,5 pro 1000 Patiententagen auf Normalstationen im Vergleich zu 19,4 auf Intensivstationen und 58 % der Patientinnen und Patienten an der Intensivstation waren zumindest einer potenziellen Arzneimittelinteraktion ausgesetzt [3, 17].

Bei der Bewertung des Risikos für Arzneimittelinteraktionen und damit verbundene unerwünschte Arzneimittelwirkungen (UAW) spielen sowohl patienten- als auch substanzspezifische Faktoren eine entscheidende Rolle. Substanzen mit einem engen therapeutischen Fenster stellen ein besonderes Risiko dar.

Auf der Ebene der Substanzen ist es entscheidend, verschiedene Medikamente mit Interaktionspotenzial für Cytochrom-P450-(CYP)-bezogene pharmakokinetische Wechselwirkungen zu berücksichtigen. Das CYP-Enzymsystem spielt eine zentrale Rolle im Arzneimittelstoffwechsel und kann die Metabolisierung von Medikamenten erheblich beeinflussen (Tab. 1). Die pharmakokinetischen Interaktionen zwischen Medikamenten auf der Ebene der CYP-Enzyme können zu veränderten Metabolisierungsraten führen, was wiederum die Plasmakonzentrationen und die Wirksamkeit der beteiligten Arzneimittel beeinflusst. Dies kann sowohl zu einer erhöhten Toxizität als auch zu einem verminderten therapeutischen Effekt führen.

**Tab. 1:** Inhibitoren (nach Stärke) und Induktoren von Cytochrom-P450-(*CYP*)-Isoenzymen laut Fachinformation der Medikamentenhersteller

CYP-Isoenzym	Schwache Inhibitoren	Moderate Inhibitoren	Starke Inhibitoren	Induktoren
CYP1A2	Citalopram, Efavirenz	Vemurafenib, Rucaparib	Ciprofloxacin, Fluvoxamin	Rauchen (Tabak), Rifampicin
CYP2C9	Voriconazol	Fluconazol, Metronidazol, Amiodaron	Sulfaphenazol	Rifampicin, Carbamazepin, Enzalutamid, Johanniskraut
CYP2C19	Cimetidin, Citalopram	Omeprazol, Fluoxetin, Esomeprazol	Fluvoxamin, Ticlopidin	Rifampicin, Efavirenz, Johanniskraut
CYP2D6	Diphenhydramin, Escitalopram	Abirateron, Clobazam, Halofantrin	Chinidin, Fluoxetin, Paroxetin	–
CYP3A4	Atomoxetin, Esomeprazol, Ivacaftor	Aprepitant, Ciprofloxacin, Crizotinib, Fluconazol	Clarithromycin, Ketoconazol, Itraconazol, Ritonavir, Saquinavir, Telaprevir, Idelalisib	Rifampicin, Carbamazepin, Johanniskraut, Dabrafenib, Efavirenz

Inhibitoren konkurrieren mit anderen Medikamenten um ein bestimmtes Enzym und beeinflussen somit den Stoffwechsel des Substratmedikaments. Dies führt in der Regel zu erhöhten Wirkstoffkonzentrationen. Im Fall einer inaktiven Vorstufe eines Medikaments (Prodrug) beeinträchtigt der Inhibitor jedoch die Aktivierung der Substanz, wodurch das Medikament unwirksam wird. Die Stärke eines Inhibitors wird anhand seiner Fähigkeit gemessen, die Fläche unter der Plasmakonzentration-Zeit-Kurve (AUC) zu erhöhen oder die Clearance eines Substratmedikaments zu reduzieren. Ein starker Inhibitor verursacht eine ≥ 5-fache Zunahme der AUC oder eine Reduzierung der Clearance um mehr als 80 %. Ein mäßiger Inhibitor bewirkt eine 2‑fache bis < 5-fache Zunahme der AUC oder eine Reduktion der Clearance um 50–80 %. Ein schwacher Inhibitor induziert eine ≥ 1,25-fache, aber < 2-fache Zunahme der AUC oder eine Reduzierung der Clearance um 20–50 %. Bei einigen Substanzen gibt es nur In-vitro-Evidenz für ihre inhibitorischen Eigenschaften.

Der Beginn des Effekts tritt innerhalb von Minuten bis Stunden nach der Verabreichung eines Inhibitors oder innerhalb von 3–5 Tagen nach Beginn eines Induktors ein. Nach dem Absetzen des beeinflussenden Medikaments wirkt ein reversibler Inhibitor abhängig von seiner Halbwertszeit nach, während die Wirkdauer eines irreversiblen Inhibitors von der Neubildung des inhibierten Enzyms abhängt. Die Induktion dauert etwa 3–5 Tage nach Absetzen des Induktors an und basiert auf der Halbwertszeit des induzierten Enzyms.

Patientenspezifische Faktoren können das Risiko für gefährliche Arzneimittelinteraktionen erhöhen

Patientenspezifische Faktoren, wie Alter, Geschlecht, genetische Variationen im Arzneimittelstoffwechsel, Begleiterkrankungen und die gleichzeitige Anwendung anderer Medikamente, können das Risiko für potenziell gefährliche Arzneimittelinteraktionen erheblich erhöhen [15, 30]. Einige Interaktionen, die speziell bei kritisch Kranken auftreten, sind in Tab. 2 dargestellt.

**Tab. 2:** Spezifische Interaktionen mit daraus resultierendem Risiko laut Fachinformation der Hersteller [3, 41]

CYP(3A4)-Hemmer	Substrate	Risiken
Amiodaron	Phenprocoumon	Blutung
Clarithromycin	Atorvastatin, Simvastatin	Erhöhte Kreatinkinase
Erythromycin	Cyclosporin, Tacrolimus	Nierenprobleme, ZNS
Verapamil	Everolimus, Sirolimus	Bradykardie
Diltiazem	Midazolam	Erhöhte Sedation
Itraconazol	Calciumantagonisten	Hypotonie, Herzfrequenz↓
Voriconazol	Carbamazepin	ZNS-Depression
Fluconazol	Phenytoin	ZNS-Depression
Ritonavir (Proteaseinhibitor)	Methadon	Verlängerung der QTc-Zeit
Andere Proteaseinhibitoren	Opiate (Fentanyl, Buprenorphin, Oxycodon, Tramadol)	ZNS-Depression
*CYP-Induktoren*	*Substrate*	*Risiken*
Rifampicin	Phenprocoumon	Thrombusbildung
Bosentan	Cyclosporin, Tacrolimus	Abstoßung
Carbamazepin	Mycophenolatmofetil (MMF, Mycophenolsäure)	Abstoßungsreaktion
Phenytoin	Opiate	Analgesie↓
Phenobarbital	Kalziumantagonisten	Bluthochdruck↑
*Andere wichtige Interaktionen*		
Cyclosporin + Statine	–	Erhöhtes Risiko für Rhabdomyolyse, niedrigste Dosis wählen; (Kontraindikation: Rosuvastatin, günstig: Fluvastatin)
Carbapeneme + Valproat	–	Valproatspiegelabfall innerhalb von 24 h, andere Antibiotikaklasse wählen
Protonenpumpeninhibitor + Itraconazol	–	Verminderte Resorption von Itraconazolkapseln (bei pH-Wert-Erhöhung), Itraconazollösung verwenden, ev. Spiegelkontrolle
Ibuprofen + Azetylsalizylsäure	–	Ibuprofen 1–2 h nach Azetylsalizylsäure verabreichen, um eine verminderte Azetylsalizylsäurewirkung zu vermeiden
Ritonavir + Methadon	–	Ritonavir induziert langfristig CYP2C9, -19 und Uridindiphosphatglucuronyltransferase, Abfall der Methadonspiegel, cave: Entzug
Linezolid/Serotoninwiederaufnahmeinhibitor/Tramadol	–	Erhöhtes Risiko für Serotoninsyndrom in Kombination

*CYP* Cytochrom-P-450

### Verlängerter Krankenhausaufenthalt und erhöhte Mortalität

In einer systematischen Übersicht wurde festgestellt, dass 58 % der Patientinnen und Patienten auf Intensivstationen, und damit doppelt so viele wie auf allgemeinen Stationen, potenziellen Arzneimittelwechselwirkungen ausgesetzt sind [17, 37]. Diese Wechselwirkungen können zu unerwünschten Arzneimittelereignissen führen, die mit einer erhöhten Mortalität, Morbidität und Krankenhauskosten verbunden sind [21]. Die Berücksichtigung der klinischen Relevanz von potenziellen Interaktionen im Intensivstationsumfeld ist wichtig, da nur die Hälfte dieser festgestellten Interaktionen als klinisch relevant erachtet wurde [1].

Bei einer weiteren Studie war trotz der hohen Prävalenz potenzieller Arzneimittelwechselwirkungen auf Intensivstationen ihre klinische Relevanz im Allgemeinen gering [32]. Darüber hinaus zeigten die Daten eine Diskrepanz zwischen verschiedenen Interaktionsdatenbanken [32].

## Substanzen mit hohem Interaktionsrisiko

Häufig eingesetzte Substanzen, die aufgrund ihrer pharmakokinetischen oder pharmakodynamischen Eigenschaften ein besonders hohes Interaktionspotenzial haben, sind immer wieder in intensivmedizinisch relevante Wechselwirkungen involviert. Einige davon seien hier als Beispiele herausgegriffen.

### Amiodaron

Das Klasse-III-Antiarrhythmikum Amiodaron zeichnet sich durch seine sehr lange Plasmahalbwertszeit von 25–100 Tagen aus und hemmt die CYP-Isoenzyme 2A6, 2C9, 2D6, 3A4 und das Transporterprotein P-Glykoprotein (P-gp), auch als MDR1 oder ABCB1-Protein bezeichnet. Die Kombination von Amiodaron mit oralen Antikoagulanzien – direkt wirkenden und Vitamin-K-Antagonisten – führt zu erhöhten Antikoagulanzienspiegeln im Plasma und damit zu einem gesteigerten Blutungsrisiko [20, 33, 35]. Über eine tödliche Hirnblutung während der Behandlung mit Rivaroxaban und Amiodaron bei neu aufgetretenem Vorhofflimmern wurde berichtet [36].

Außerdem werden die Plasmakonzentrationen und damit die sedierende Wirkung von Midazolam, Fentanyl, Sufentanil und Phenytoin erhöht, ebenso wie die Wirkung der Immunsuppressiva Cyclosporin, Sirolimus und Tacrolimus, was mit Infektionen, Neurotoxizität und Nephrotoxizität einhergehen kann. Pharmakodynamische Wechselwirkungen mit Amiodaron können sich einerseits in einer Verstärkung der bradykardisierenden Wirkung unter begleitender Gabe von β‑Blockern oder Digitalis manifestieren, andererseits in Form einer QTc-Zeit-Verlängerung bei Kombination mit anderen QTc-Zeit-verlängernden Substanzen, wie mit Neuroleptika, Antidepressiva, einigen Antibiotika wie Makroliden oder Azolantimykotika [29].

Die Statine Simvastatin und Atorvastatin sind Substrate des Isoenzyms CYP3A4 und sollen daher wegen der Gefahr einer Rhabdomyolyse nicht mit Amiodaron kombiniert werden [14, 22].

### Dexamethason

Dexamethason findet bei kritisch Kranken eine breite Anwendung, so bei hämatoonkologischen und neurologischen Erkrankung, aber auch bei COVID-19. Dexamethason induziert CYP3A4, CYP2C9 und P‑gp sowie in geringerem Ausmaß auch CYP2C8 und CYP2B6. Dementsprechend wurden unter Dexamethason subtherapeutische Voriconazolspiegel gemessen, während die Wirkung auf die Konzentrationen direkt wirkender Antikoagulanzien offenbar gering ist [31]. Andererseits ist Dexamethason ein CYP3A4-Substrat und kann somit unter gleichzeitiger Behandlung mit CYP3A4-Inhibitoren, z. B. dem Antiemetikum Aprepitant, akkumulieren oder durch Anwendung eines CYP3A4-Induktors, wie Phenobarbital, Carbamazepin oder Rifampicin, verstärkt metabolisiert und damit in seiner Wirkung abgeschwächt werden [8].

### Analgetika und Sedativa

Morphin ist ein Substrat der Uridindiphosphat(UDP)-Glucuronyltransferase (UGT). Durch Hemmung dieses Enzyms kann es zu einer verstärkten Wirkung und zu Nebenwirkungen von Morphin, wie Vigilanzminderung, Ventilationsstörung, Übelkeit und Obstipation, kommen. Eine Entwöhnung von der Beatmung kann dadurch verzögert werden. Dies gilt analog auch für Hydromorphon. Buprenorphin, Fentanyl und Sufentanil werden mittels CYP3A4 transformiert und können bei begleitender Behandlung mit einem CYP3A4-Inhibitor wie z. B. Amiodaron oder einem Azolantimykotikum akkumulieren.

Midazolam wird ebenfalls über CYP3A4 metabolisiert, was zu einer verlängerten Wirkung bei begleitender Gabe von CYP3A4-Inhibitoren, wie Azolantimykotika, Erythromycin, Clarithromycin oder Amiodaron, führt. CYP3A4-Induktoren bewirken hingegen erniedrigte Konzentrationen.

Lorazepam hat eine längere Plasmahalbwertszeit als Midazolam, wodurch die Substanz grundsätzlich weniger gut steuerbar ist. Es wird mittels UGT transformiert. Pharmakokinetische Interaktionen spielen bei Sedierung mit Lorazepam aber eine geringe Rolle [13].

Propofol wird ebenfalls in der Leber metabolisiert. Beteiligt sind UGT1A9, CYP2B6 und CYP2C9, für die unterschiedlich aktive Allele existieren [10]. Gefürchtete Nebenwirkungen von Propofol sind v. a. eine Verlängerung der QTc-Zeit, Blutdruckabfälle bis zum Herz-Kreislauf-Stillstand und das Propofolinfusionssyndrom (PRIS), das mit Rhabdomyolyse, Laktatacidose und Multiorganversagen einhergehen kann. Das PRIS tritt offenbar vermehrt unter hochdosierter Noradrenalintherapie auf [34, 38].

### Antiinfektiva

#### Antibiotika

Antibiotika mit einem hohen Potenzial für relevante pharmakokinetische Interaktionen sind die Makrolide Erythromycin und Clarithromycin, das Chinolon Ciprofloxacin und Rifampicin [11]. Erythromycin und Clarithromycin inhibieren CYP3A4 und P‑gp, Erythromycin zusätzlich CYP1A2 [25]. Azithromycin ist nicht in pharmakokinetische Interaktionen involviert, verlängert jedoch, wie alle Makrolide, Chinolone und Metronidazol, die QTc-Zeit und erhöht damit das Risiko für Torsade-de-Pointes-Tachykardien. Ciprofloxacin ist ein CYP1A2-Inhibitor, der zu einer Konzentrationserhöhung von CYP1A2-Substraten, wie Propranolol, Theophyllin und Fluvoxamin, führen kann.

Rifampicin ist ein starker Induktor verschiedener CYP-Isoenzyme

Rifampicin ist ein starker Induktor der Isoenzyme CPY2B6, 2C8, 2C9, 2C19 und 3A4 sowie der Transporter P‑gp und OATP1 („organic anion transport protein 1“) und ein schwacher Induktor der UGT1A1. Damit führt Rifampicin zum verstärkten Abbau und zum Wirkungsverlust zahlreicher Medikamente, darunter Azolantimykotika, Antikoagulanzien, Makrolide, aber auch der Immunsuppressiva Cyclosporin, Tacrolimus und Sirolimus.

Linezolid ist ein reversibler, nichtselektiver Hemmer der Monoaminooxidase (MAO), die die Oxydation von Serotonin, aber auch Noradrenalin und Adrenalin katalysiert. Diese Hemmung kann bis zu etwa 2 Wochen nach Absetzen von Linezolid andauern und bei Kombination mit Serotoninwiederaufnahmeinhibitoren (SSRI), trizyklischen Antidepressiva, 5‑HT1-Serotonin-Rezeptoragonisten (Triptanen), direkt oder indirekt wirkenden Sympathomimetika, dopaminergen Wirkstoffen, Morphin und Opioiden zu einem Serotoninsyndrom führen. Typische Symptome sind Blutdruckanstieg, Fieber, Tremor, Hyperreflexie, verstärkte Darmmotilität, Mydriasis, Hautrötung, Unruhe und Verwirrtheit [9].

Aufgrund ihrer pharmakokinetischen Eigenschaften haben β‑Laktam-Antibiotika ein geringes Interaktionspotenzial. Flucloxacillin kann allerdings die Exposition gegenüber den Azolantimykotika Fluconazol, Voriconazol, Posaconazol und Isavuconazol und damit deren Wirksamkeit reduzieren. Als Mechanismus wird eine Induktion relevanter CYP-Isoenzyme sowie von UGT und P‑gp diskutiert [11]. Carbapeneme (Ertapenem, Imipenem und Meropenem) führen zu verringerten Plasmaspiegeln von Valproinsäure und reduzieren damit deren antikonvulsive Wirksamkeit. Der zugrunde liegende Mechanismus ist unklar [11]. Andererseits wurden unter hochdosierter Meropenemtherapie erhöhte Voriconazolspiegel bei vermindertem Abbau über CYP2C19 und 3A4 gemessen [26].

Aminoglykoside sind aufgrund ihrer Toxizität heute nur noch selten indiziert. Eine Kombination mit anderen nierentoxischen Substanzen wie Immunsuppressiva verstärkt ihre Nephrotoxizität.

#### Antimykotika

Eine ausgeprägte Nephrotoxizität kennzeichnet auch das Breitbandantimykotikum Amphotericin B, das heute fast ausschließlich als weniger toxische liposomale Formulierung eingesetzt wird. Amphotericin B ist an keinen bekannten pharmakokinetischen Interaktionen beteiligt, verstärkt aber die Wirkung nephrotoxischer und myelotoxischer Begleitmedikationen [7].

Flucytosin ist in Kombination mit Amphotericin B Mittel der Wahl bei schwerer Kryptokokkose und bei kardialen und zerebralen Candida-Infektionen. Seine Aufnahme in die Pilzzelle wird durch Cytarabin gehemmt, was die Umwandlung in das wirksame Fluorouracil verhindert. Nukleosidanaloga, wie Brivudin und Sorivudin, hemmen hingegen die Dihydropyrimidindehydrogenase und damit den Abbau auch noch bis zu 4 Wochen nach ihrem Absetzen. Immunsuppressiva und antineoplastisch wirksame Chemotherapeutika erhöhen die Myelotoxizität von Flucytosin [7].

Azole sind die Antimykotika mit dem größten Potenzial pharmakokinetischer Interaktionen

Die Antimykotika mit dem größten Potenzial pharmakokinetischer Interaktionen sind die Azole. Fluconazol wird renal ausgeschieden, inhibiert aber CYP2C9 und CYP3A4. Die aspergilluswirksamen Breitbandazole werden in der Leber metabolisiert und sind Substrate für CYP3A4 (Voriconazol, Isavuconazol), CPY2C9 und CPY2C19 (Voriconazol) sowie für UGT und P‑gp (Posaconazol). Andererseits sind Azolantimykotika Inhibitoren der CYP-Isoenzyme CYP2C9, 2C19 und 3A4 sowie der UGT und der Transporter BCRP, OCT2 und P‑gp. Erhöhte Plasmaspiegel und eine verstärkte, länger anhaltende Wirkung von Immunsuppressiva, Benzodiazepinen, oralen Antikoagulanzien und Antihistaminika sind daher zu beachten. Immunsuppressiva sind bei einer Pilzinfektion möglichst vorrübergehend zu pausieren oder ihre Dosis ist unter engmaschiger Spiegelkontrolle zu reduzieren. Tyrosinkinaseinhibitoren, wie Ibrutinib, Lorlatinib und Zanubrutinib, die CYP3A4-Substrate sind, zeigen unter Komedikation mit Azolen ebenfalls stark erhöhte Spiegel. Die jeweilige Fachinformation empfiehlt eine Dosisreduktion, wenn Tyrosinkinaseinhibitoren unter Azolbehandlung weiter verordnet werden müssen.

Alle Azolantimykotika mit Ausnahme von Isavuconazol können die QTc-Zeit verlängern. Aufgrund ihrer komplexen Pharmakokinetik müssen die Wirkspiegel der Azole regelmäßig kontrolliert werden, um eine wirksame Konzentration sicherzustellen und Toxizität zu vermeiden. Bei Voriconazol ist eine besonders variable, nichtlineare Pharmakokinetik zu beachten.

Die Echinocandine weisen ein weit geringeres Interaktionspotenzial auf als die Azole. Anidulafungin wird im Plasma durch eine spontanen Ringöffnung degradiert. Nach Phase-I- und Phase-II-unabhängiger Hydrolyse und Azetylierung wird es biliär eliminiert. Klinsch relevante Arzneimittelinteraktion mit Anidulafungin sind bisher nicht bekannt.

Obwohl Caspofungin in vitro mit Transporterproteinen interferiert, sind die angeführten Wechselwirkungen klinisch unbedeutend. Micafungin hemmt die Aktivität einiger Transporterproteine, v. a. Multidrug-resitance-Protein 4 (MRP-4) und in geringem Ausmaß auch CYP3A4. Die sich daraus ergebenden pharmakokinetischen Interaktionen sind aber von geringer Bedeutung. Zu beachten ist hingegen die potenzielle Hepatotoxizität von Micafungin, vor der die Fachinformation warnt.

#### Virostatika

Für die virostatische Behandlung von COVID-19 stehen die oral verfügbaren Kombination von Nirmatrelvir und Ritonavir sowie das intravenös zu applizierende Remdesivir zur Verfügung. Obwohl Nirmatrelvir-Ritonavir vorwiegend für die ambulante Behandlung eingesetzt wird, sei auf sein komplexes Interaktionsprofil hingewiesen. Ritonavir dient aufgrund seiner hemmenden Wirkung auf CYP3A4 als „Booster“ für Nirmatrelvir, indem es dessen Abbau verzögert. Allerding induziert es die Enzyme CYP1A2, CYP2B8, CYP2C9, CYP2C19 und UGT, was zur verstärkten Metabolisierung begleitend verabreichter Substrate führen kann. Klinisch relevant ist jedoch die Spiegelerhöhung von CYP3A4-Substraten, wie oraler Antikoagulanzien, der Statine Simvastatin und Atorvastatin, von Kalziumantagonisten, Immunsuppressiva und Amiodaron. Sofern Nirmatrelvir-Ritonavir dennoch während einer Therapie mit einem dieser Substrate verordnet wird, ist diese für 8 Tage zu pausieren [27].

Remdesivir hat für die Behandlung Schwerkranker einen höheren Stellenwert

Remdesivir hat für die Behandlung Schwerkranker einen höheren Stellenwert. In vitro ist es ein Substrat für Plasma- und Gewebeesterasen, für CYP3A4, für den organischen Anionentransporter („organic anion-transporting polypeptide“) 1B1 (OATP1B1) und für P‑gp. Der Remdesivirmetabolit GS-704277 ist ein Substrat für OATP1B1 und OATP1B3. In vitro hemmt Remdesivir CYP3A4, UGT1A1, MATE1, OAT3, OCT1, OATP1B1 und OATP1B3 [19]. Aufgrund des Fehlens klinischer Daten kann die Relevanz daraus resultierender Interaktionen mit Remdesivir derzeit kaum beurteilt werden. Seine bradykardisierende Wirkung ist im Sinne möglicher pharmakodynamischer Interaktionen in Betracht zu ziehen. Im Vergleich mit Nirmatrelvir-Ritonavir dürfte das Potenzial für klinisch relevante Interaktionen von Remdesivir wohl geringer sein.

Bei der Behandlung der Zytomegalievirusinfektion mit Ganciclovir sind pharmakodynamische Interaktionen zu berücksichtigen, so eine erhöhte Krampfneigung in Kombination mit Imipenem-Cilastatin. Die Nephrotoxizität und die Myelotoxizität können durch die Gabe anderer nephrotoxischer Pharmaka, wie Amphotericin‑B, Trimethoprim-Sulfamethoxazol oder Pentamidin, bzw. myelotoxischer Substanzen, wie Flucytosin, Vincristin oder Vinblastin, verstärkt werden.

Aciclovir wird in unveränderter Form renal ausgeschieden, weshalb die Dosis der Nierenfunktion anzupassen ist. Der Hersteller warnt vor erhöhten Lithiumspiegel bei Kombination mit hochdosiertem Aciclovir.

## Strategien zur Vermeidung unerwünschter Arzneimittelwechselwirkungen

### Einsatz von elektronischen Warnsystemen und Interaktionsprogrammen

Computergestützte ärztliche Anordnungserfassung bezeichnet eine Vielzahl computerbasierter Systeme, die den Medikamentenverschreibungsprozess erleichtern sollen. Diese Systeme werden durch die Integration von klinischen Entscheidungsunterstützungssystemen weiter verbessert. Sieumfassen interaktive Software, die Gesundheitsfachkräfte bei klinischen Entscheidungen unterstützt. Diese Prozesse zielen auf die Verschreibungsphase ab und sollen damit die Medikamentensicherheit auf verschiedene Weise wie folgt verbessern:

- Standardisierung der klinischen Praxis;
- Verbesserung der Vollständigkeit und Lesbarkeit von Einträgen;
- Benachrichtigung über Medikamentenallergien, *Arzneimittelinteraktionen* und kumulative Dosisgrenzen;
- Aktualisierung mit den neuesten Medikamenteninformationen;
- Dosierungsanpassungen basierend auf Patientenmerkmalen;
- zeitnahe Kommunikation kritischer Veränderungen im Zustand eines Patienten/einer Patientin, die wiederum angemessene Anpassungen erleichtert.

Weitere Onlineprogramme sind verfügbar, die Medikamenteninteraktionen klassifizieren und nach Schweregrade gruppieren. Manche Programme zeigen auch Empfehlungen für die klinische Praxis an (Tab. 3).

**Tab. 3:** Arzneimittelinteraktionsprogramme

Interaktionsprogramm	Beschreibung	Link
Lexicomp^TM^ (Wolters Kluwer, Alphen aan den Rijn, Niederlande) (kostenpflichtig)	Ein umfassendes Programm zur Überprüfung von Arzneimittelinteraktionen, das eine umfangreiche Datenbank von Medikamenten und deren potenziellen Interaktionen bietet	https://online.lexi.com/lco/action/login
Micromedex Drug Interactions (Merative, Ann Arbor, MI, USA) (kostenpflichtig)	Ein weiteres Programm mit einer umfassenden Datenbank von Arzneimittelinteraktionen, das von medizinischen Fachleuten häufig für die Interaktionsprüfung verwendet wird	https://www.micromedexsolutions.com/
Epocrates Drug Interactions (Epocrates, San Mateo, CA, USA) (Login erforderlich)	Eine mobile Anwendung, die von Ärzten und medizinischem Personal genutzt wird, um schnell und einfach nach Arzneimittelinteraktionen zu suchen	https://www.epocrates.com/online/interaction-check
Medscape Drug Interaction Checker (frei zugänglich)	Ein Onlinetool, das von Gesundheitsdienstleistern verwendet wird, um potenzielle Wechselwirkungen zwischen Arzneimitteln zu überprüfen und zu bewerten	https://reference.medscape.com/drug-interactionchecker
Offizielle Schweizer Fachinformationen (frei zugänglich)	Schweizer Fachinformationen zu Arzneimitteln, die offizielle Angaben zu Interaktionen bieten	https://www.swissmedicinfo.ch/
Interaktionscheck basierend auf Schweizer Fachinformationen (frei zugänglich)	Ein Interaktionscheck-Tool, das auf den Schweizer Fachinformationen basiert und eine umfassende Überprüfung von Arzneimittelinteraktionen ermöglicht	https://compendium.ch/
Qualitätszentrum für Medikamentensicherheit, Psychiatrische Dienste Aargau (15 Tage gratis)	Ein Zentrum für Medikamentensicherheit, das Informationen und Ressourcen zur Sicherheit von Medikamenten bereitstellt, insbesondere im Bereich der Psychiatrie	https://www.mediq.ch/
Onlinemedikamenteninformation mit Interaction Check	Drugs.com bietet unabhängige Informationen zu mehr als 24.000 verschreibungspflichtigen Medikamenten, rezeptfreien Medikamenten und Naturprodukten sowie ebenfalls einen Interaktionscheck	https://www.drugs.com/drug_interactions.html
Medscape Medikamentenupdate	Eine Ressource von Medscape, die regelmäßige Updates und Informationen zu Medikamenten, einschließlich potenzieller Interaktionen, bereitstellt	https://reference.medscape.com/drug-interactionchecker
HIV-Medikamentencheck	Ein spezialisiertes Tool zur Überprüfung von Arzneimittelinteraktionen bei HIV-Medikamenten, das Informationen und Ratschläge zur sicheren Verwendung von HIV-Medikamenten bietet	https://hiv-druginteractions.org/
Liverpool Hep Drug Interaction Checker	Interaktionstool der Universität Liverpool	https://www.hep-druginteractions.org/
WebMD Interaction Checker	Drug Interaction Checker Tool, um potenziell schädliche und unsichere Kombinationen von verschreibungspflichtigen Medikamenten zu finden und zu identifizieren	https://www.webmd.com/interaction-checker/
CYP450-Interaktionstabelle	Substrate, Inhibitoren und Induktoren von CYP450 von der Indiana University	https://drug-interactions.medicine.iu.edu/MainTable.aspx

*CYP* Cytochrom-P-450

Es ist zu beachten, dass diese Programme nicht speziell für kritisch Kranke entwickelt wurden. Auf der Intensivstation werden ungefähr 90 % der potenziellen Interaktionswarnungen außer Kraft gesetzt und 84 % dieser Außerkraftsetzungen scheinen angemessen zu sein, da die Warnungen geringfügig sind [39, 40]. Daher könnte die Anpassung von Prozessen an die spezifischen Gegebenheiten der Intensivstation die Alarmmüdigkeit verringern und die Medikamentensicherheit bei kritisch Kranken verbessern. Eine neue multizentrischen Studie untersuchte den Einfluss der Anpassung von potenziellen Arzneimittelinteraktionswarnungen an die Intensivstationsbedingungen auf die Häufigkeit der Verabreichung von Hochrisikomedikamentenkombinationen [3]. In der Studie wurden nur Warnungen für potenzielle Arzneimittelinteraktionen von hoher Relevanz geliefert. Diese Intervention wurde auf der Ebene der Intensivstation durchgeführt und richtete sich an Ärztinnen und Ärzte. Insgesamt wurden 10.423 kritisch Kranke bewertet, wobei 9887 Patientinnen und Patienten eingeschlossen wurden. Die Ergebnisse zeigten, dass die Anpassung von potenziellen Interaktionswarnungen an die Bedingungen auf der Intensivstation zu einer signifikanten Verringerung der Anzahl von verabreichten Hochrisikomedikamentenkombinationen um 12–14 % führte. Dies ging auch mit einer Verringerung der Aufenthaltsdauer auf der Intensivstation und mit einer Zunahme der angemessen überwachten Hochrisikomedikamentenkombinationen einher. Die Ergebnisse dieser Studie legen nahe, dass die Anpassung von Warnungen an die Intensivstationsbedingungen die Verabreichung von Hochrisikomedikamentenkombinationen signifikant verringern kann.

### Interdisziplinäre Zusammenarbeit

Zudem ist eine effektive Zusammenarbeit zwischen Spezialisten, wie Intensivmedizinern, Hämatoonkologen, Infektiologen, klinischen Pharmakologen und anderen Fachkräften, von entscheidender Bedeutung, um die Medikamentensicherheit und -effektivität bei kritisch Kranken zu gewährleisten. Eine enge Kooperation zwischen diesen Spezialisten ermöglicht es, komplexe medikamentöse Therapien zu optimieren, potenzielle Risiken zu identifizieren und geeignete Strategien zur Vermeidung von Arzneimittelinteraktionen und unerwünschten Arzneimittelereignissen zu entwickeln. Durch den Austausch von Fachwissen und die gemeinsame Entscheidungsfindung können sie dazu beitragen, die bestmögliche Behandlung für kritisch Kranke zu gewährleisten und deren Sicherheit zu verbessern.

## Fazit für die Praxis

- Arzneimittelinteraktionen können Toxizität oder Wirkungsverlust der medikamentösen Therapie verursachen.
- Amiodaron, Antikoagulanzien, bestimmte Antiinfektiva, Immunsuppressiva und einige ZNS-wirksame Substanzen sind besonders häufig in Interaktionen involviert.
- Strenge Risiko-Nutzen-Abwägung, therapeutisches Drugmonitoring und die Verwendung elektronischer Alert-Systeme und Datenbanken reduzieren das Risiko unerwünschter Arzneimittelinteraktionen.
